# DNA methylation in melanoma immunotherapy: mechanisms and therapeutic opportunities

**DOI:** 10.1186/s13148-025-01865-5

**Published:** 2025-04-30

**Authors:** Maya G. Deshmukh, Veronica T. Brooks, Simon F. Roy, Simon Milette, Marcus Bosenberg, Goran Micevic

**Affiliations:** 1 Medical Scientist Training Program (MD-PhD), Yale School of Medicine, New Haven, CT 06520 USA; 2 Department of Immunobiology, Yale School of Medicine, New Haven, CT 06520 USA; 3 Department of Pathology, Yale School of Medicine, New Haven, CT 06520 USA; 4 Department of Dermatology, Yale School of Medicine, New Haven, CT 06520 USA; 5 Yale Cancer Center, Yale School of Medicine, New Haven, CT 06520 USA; 6 Yale Center for Immuno-Oncology, Yale School of Medicine, New Haven, CT 06520 USA

## Abstract

Abnormal DNA methylation is a hallmark of cancer and a nearly universal feature of melanoma. DNA methylation plays well-appreciated melanoma cell-intrinsic roles, including silencing tumor-suppressor genes, regulating genomic stability, deregulating expression of oncogenes to potentiate proliferative signaling and tumor migration. With the recent success of immunological therapies for melanoma, important roles for DNA methylation are also emerging at the interface between melanoma and immune cells with the potential to regulate the anti-tumor immune response. These newly recognized roles for DNA methylation in controlling melanoma cell immunogenicity, expression of MHC and immune checkpoint molecules as well as T cell phenotypes in the tumor microenvironment raise the possibility of using DNA methylation to develop improved therapies and methylation-based biomarkers. In addition to reviewing the “immune dimension” of DNA methylation, we summarize recent developments with potential clinical applications in melanoma, such as targeted DNA methylation editing, single-cell methylation approaches, and measurement of circulating methylated DNA. An improved understanding of the immune roles of DNA methylation presents an exciting opportunity for continued improvement of care and outcomes for patients with melanoma.

## Background

The development of melanoma is associated with abnormal patterns of hyper- and hypo- DNA methylation and histone acetylation and methylation [1–4]. DNA hypermethylation and resulting silencing at the promoter of the tumor-suppressor *PTEN* has been detected in more than half of melanomas even in the absence of *PTEN* gene deletions, and *CDKN2A* promoter methylation has been reported in a quarter of cutaneous melanoma metastases [3, 5, 6]. Frequent differential methylation of genes associated with oncogenesis, DNA repair, apoptosis, metastasis, and differentiation have been reported in melanoma and are reviewed elsewhere [3].

Epigenetic alterations also impact the immunogenicity of tumor cells and the phenotypic trajectories of immune cells involved in the anti-tumor response. Understanding the landscape of epigenetic changes in tumor and immune cells in melanoma progression and therapy will provide valuable insights into potential pharmacologic interventions to modulate the epigenome pharmacologically. Here, we summarize recent insights into how DNA methylation in melanoma may impact tumor immunogenicity and outline future directions for the development of novel epigenome-targeting immunotherapies for melanoma. In this review, the following aspects of melanoma-associated DNA methylation will be discussed: (I) epigenetic alterations and melanoma-intrinsic immunogenicity, (II) epigenetic regulation of immune cells in the anti-tumor response, (III) DNA methylation as a prognostic biomarker for immunotherapy response, and (IV) emerging technologies in melanoma methylation research.

## Epigenetic alterations and melanoma-intrinsic immunogenicity

The development of targeted therapies and immune checkpoint blockade has transformed the therapeutic landscape of melanoma, doubling the 5-year relative survival for distant-stage melanoma between 2009 and 2015 [7]. Despite these continuing advances, 5-year overall survival for advanced melanoma treated with dual immune checkpoint blockade currently remains around 50% [8]. These data point to the need for alternative classes of therapies, combinations of therapies, and prognostic biomarkers to improve clinical outcomes.

Tumors that fail to respond to immunotherapy are considered immunologically “cold.” Cold tumors typically possess some combination of low mutational burden, lack of genomic instability and an immunosuppressive tumor microenvironment. There is an unmet need to induce immune recognition in otherwise cold tumors to broaden the scope of immunotherapy responses. There are multiple strategies for generating an immune response within tumors. Epigenetic modifiers, particularly DNA-methylating enzymes, have long been recognized as a central regulators of melanoma immunogenicity that can be targeted to reinvigorate anti-tumor immune responses.

DNA methylation and histone modification are carried out by distinct enzymatic complexes and generally orchestrate silencing of noncoding DNA regions, but it is important to consider the temporal association of DNA methylation and histone modification in the context of epigenetic targeting. For example, there is evidence that functional gene silencing via the accumulation of nucleosomes in a regulatory region of a gene may be a prerequisite for de novo methylation, suggesting that high nucleosome occupancy at baseline may in some cases precede DNA methylation [9, 10]. DNA methylation patterns are thus highly dependent on the local histone modification milieu [10]. For example, activating H3K4 methylation inhibits de novo methylation by DNA methyltransferase (DNMT) 3A/B, while a lack of H3K4 methylation is permissive of DNMT3A/B-mediated methylation [11]. This activity may be mediated by the catalytically inactive DNMT3L, which binds only unmethylated H3 tails via its N-terminal cysteine-rich domain and recruits DNMT3A/B for methylation [11]. Further, experiments in embryonic stem cells and embryonic carcinoma cells demonstrate differential patterns of H3K27 and H3K4 methylation and DNA hypermethylation-associated di- and tri-methylation of H3K9, respectively, suggesting specific patterns of histone modifications may in certain cases predispose sites for cancer-associated DNA hypermethylation [12, 13]. Together, these studies suggest a potential chronological link between site-specific transcriptional activity at baseline, nucleosome occupancy and histone modifications, and de novo DNA methylation which should be considered in further preclinical studies aiming to establish novel strategies for epigenome targeting for immunotherapy.

DNA methylation has long been recognized to regulate the expression of cancer testis antigens in melanoma, including the MAGE family MAGE-A1, MAGE-A3, NY-ESO-1, GAGE and SSX families which are normally silenced in somatic tissues, but are expressed in the testis and during tumorigenesis due to aberrant global hypomethylation. This phenomenon contributes to tumor immunogenicity, making CTAs attractive targets for cancer immunotherapy [14]. Several studies have demonstrated that treatment with DNMT inhibitors, such as decitabine and azacitidine, can upregulate CTA expression, thereby enhancing tumor antigenicity and sensitivity to T cell-mediated killing [10, 15]. For example, treatment with DNMT inhibitors increases *NY-ESO-1* expression in melanoma cells, promoting recognition by cytotoxic T lymphocytes [16]. However, the role of CTAs in melanoma progression remains complex. Some CTAs not only serve as immunogenic targets, but also contribute to tumor cell survival, proliferation, and resistance to therapy [17]. Preferentially expressed antigen in melanoma (PRAME) is a cancer testis antigen that can be induced by hypomethylating agents [18]. It is used clinically to support a diagnosis of melanoma [19], and as a target for CAR-T in cutaneous [20] and uveal melanoma [21]. Understanding the epigenetic regulation of CTAs provides opportunities to improve therapeutic strategies by combining epigenetic modulators with immune checkpoint inhibitors or cancer vaccines [22].

In addition to regulating antigen expression, DNA methylation also regulates antigen presentation through multiple mechanisms. Methylation of NLRC5 in melanoma [23] is associated with loss of MHC Class I genes HLA-A, HLA-C and B2M expression, which are linked to immunotherapy resistance and decreased survival. DNA methylation and silencing of the peptide transporter TAP1 can suppress antigen processing and presentation [24]. Treatment of melanoma cells with hypomethylating agents can restore NLRC5, TAP1 and MHC Class I expression to promote immune recognition, and response to checkpoint blockade [25]. Use of a DNMT1 inhibitor could partially demethylate, and lead to re-expression of the HLA-A3 gene in response to IFN-γ [26]. Enhanced constitutive expression of HLA-A1, HLA-A2 has similarly been reported in response to 5-aza-2'-deoxycytidine therapy [27] in melanoma. DNA methylation also regulates the expression of the gene encoding the class II transactivator (CIITA), and hypomethylating agents can restore cytokine-induced expression class II MHC genes in melanoma cells [28]. Combined therapy with hypomethylating agents and an HDAC inhibitor trichostatin A potentiated expression of HLA-DR, CIITA and the class II-associated invariant chain peptide (CLIP) in melanoma cells [29].

One strategy to improve the immunogenicity of melanoma cells is the induction of antiviral signatures in tumor cells. Most of the human genome is composed of putatively noncoding sequences, including repetitive elements like short and long interspersed nuclear elements (SINEs and LINEs) and endogenous retroviruses (ERVs) that contain long terminal repeat (LTR) regions [30]. Endogenous retroviruses are remnants of viral integrations that have been relatively stably passed down in human genomes. The age of integration is correlated to the method of silencing, with evolutionarily young ERVs silenced predominantly by DNA methylation and intermediate age ERVs silenced predominantly by histone modifications [30].

**Fig. 1:**
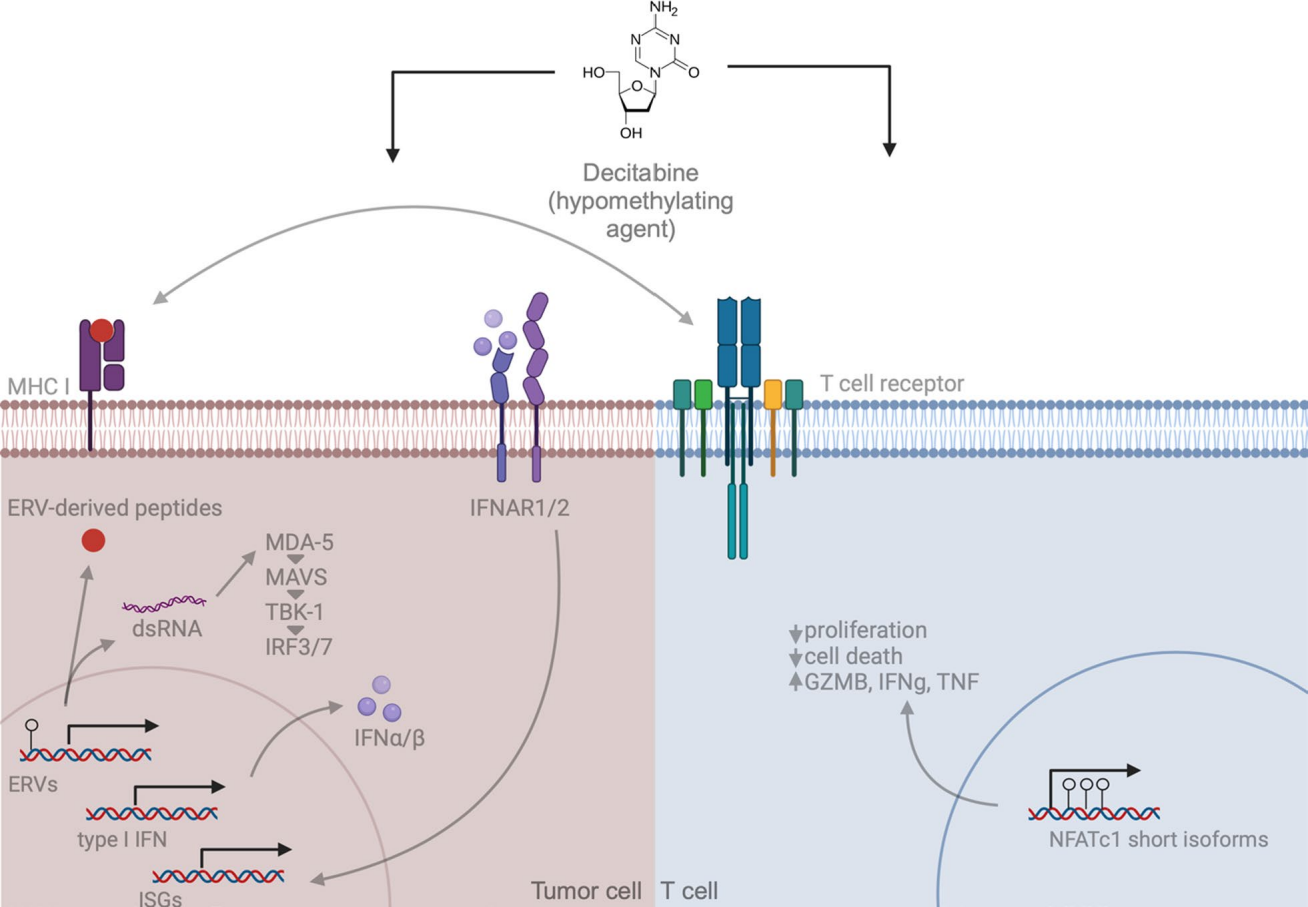
Mechanisms by which hypomethylating agents may modulate tumor cells (left) and T cells (right) in the anti-tumor immune response. The hypomethylating agent, decitabine, has activity in tumor cells and T cells in increasing immunogenicity [35, 63]. DAC treatment induces ERV expression in tumor cells, activating the MDA-5/MAVS innate immune sensing pathway, resulting in type I interferon expression [35]. Type I interferons stimulate interferon-stimulated gene (ISG) expression. ERV expression induces the production of aberrant ERV-derived peptides, which are predicted to bind to class I MHC molecules recognized by T cell receptors on CTLs. DAC treatment also impacts anti-tumor CTLs by inducing preferential expression of short isoforms of NFATc1, associated with increased CTL effector function, cytotoxicity and increased survival [63]

The reactivation of ERVs leads to a “viral mimicry” phenotype that would typically cause a pathological immune response in healthy tissues. In cancer cells, however, ERV reactivation can act as a double-edged sword. ERV reactivation and instability may lead to the activation of oncogenes or disruption of tumor suppressors, but in certain cases can also induce immune responses that sensitize tumor cells to immune recognition [31, 32]. ERV dysregulation is an emerging hallmark of several cancer types, suggesting there may be an attractive therapeutic index for targeting ERV expression as an immunosensitization strategy [33]. ERVs activate innate immune signaling via pattern recognition receptors, including toll-like receptors, members of the RIG-I family and cGAS-STING and other cytosolic DNA sensors [34]. Collectively, these receptors detect dsRNA, dsDNA, CpG DNA, ssRNA, and induce an inflammatory cellular response via expression of cytokines and upregulation of antigen presentation machinery [34]. Beyond dysregulated expression of ERVs in certain cancers at baseline, epigenetic drugs can induce their expression and promote beneficial anti-tumor inflammation. For instance, the demethylating agent 5-AZA-CdR (decitabine, or DAC) can induce ERV-derived dsRNA expression, activating the MDA-5/MAVS innate immune sensing pathway and induce expression of interferon-responsive genes and type III interferons [35] (Fig. 1), regardless of CpG island methylator phenotype (CIMP) status [35]. This suggests that ERV reactivation may be a broadly applicable strategy for immune sensitization of tumors without prior knowledge of CIMP status.

DNMT inhibitors can induce ERV-associated dsRNA and resulting type I interferon expression in melanoma, as well as several other types of cancer [22]. Expression of viral mimicry genes correlated with immunotherapy response and DNMT inhibitor treatment enhanced the response to anti-CTLA-4 therapy in a mouse model of melanoma [22]. ERV expression is regulated by several additional epigenetic mechanisms and modulation of histone modifiers can also reactivate latent endogenous retroviruses in melanoma cells in addition to modulation of DNA methylation. For instance, inhibition of the H3K4 demethylase KDM5B led to ERV reactivation, cytosolic RNA and DNA sensing, and type I interferon expression in a melanoma model in a manner dependent on the activity of the histone methyltransferase SETDB1 [36, 37]. Additional studies in other tumor models point to a shared mechanism of reactivating ERVs to sensitize tumor cells to immunotherapy by targeting both DNA methylation and histone modifications [38, 39].

## Epigenetic regulation of anti-melanoma immune responses

### Targeting immune cell DNA methylation for enhanced immunotherapy

The innate and adaptive immune systems coordinate robust initial and recall responses against foreign signatures. Innate and adaptive immune cells derive from common myeloid and lymphoid progenitors, respectively, which are derived from a common hematopoietic stem cell. Within the myeloid and lymphoid lineages, cells further differentiate into distinct mature cell types and subtypes with unique functions in the immune response. Epigenetic changes are involved in each step of differentiation that generates the immense response diversity of the immune system. The immune cells that comprise the anti-tumor response are thus targets of physiologic and systemic therapy-induced epigenetic changes. It is therefore important to consider how targeting the epigenome may impact anti-tumor immune compartments, and further, how epigenome targeting may be used specifically to favorably alter the phenotypic profiles of immune cells to potentiate the anti-tumor response.

While this section will focus primarily on the impact of DNA methylation changes in mature anti-tumor T cells, it is important to briefly highlight the role of epigenetic changes across early hematopoietic stem cell differentiation. Early fate decisions of hematopoietic stem cells (HSCs) are largely controlled by the activity of DNMTs [40]. DNMT1 activity maintains HSC self-renewal and prevents premature, restricted differentiation of HSCs into myeloerythroid progeny [41]. DNMT3A and B, on the other hand, are required for HSC differentiation [42]. Lack of both DNMT3A and B in HSCs prevents differentiation and enhances self-renewal in a B-catenin signaling-dependent manner [42]. Global methylation pattern trends bifurcate during myeloid and lymphoid lineage development. Myeloid commitment is associated with hypomethylation, whereas lymphoid commitment is associated with an increase in methylation [43]. Further myeloid differentiation steps involve dynamic fluctuations in global methylation levels and unique methylation fingerprints, but mature cells from the myeloid lineage tend to be hypomethylated relative to lymphoid-derived cells [43, 44].

Epigenetic changes are also associated with functional polarization of myeloid-derived cells. The histone deacetylase HDAC3 and histone demethylase JMJD3 have been shown to differentially control transcriptional programs responsible for M1 versus M2 macrophage polarization, respectively [45, 46]. DNMT3B plays a role in M1 polarization and resulting inflammation in the setting of obesity [47]. Different temporal patterns of H3K4 methylation have been associated with poising macrophages for faster restimulation responses in “trained” innate immune responses and programming endotoxin tolerance [48, 49]. While preclinical studies investigating the impact of systemic DNMTi therapy in combination with checkpoint blockade have been promising, it is important to consider the potential impact of epigenetic targeting therapies on progenitors and mature cells of the innate immune system that shape the tumor microenvironment (TME).

Lymphoid progenitors differentiate into T and B cells, each of which differentiate into subtypes with different functions. Cytotoxic CD8 + T cells (CTLs) are key players in the anti-tumor response and are primarily responsible for carrying out the anti-tumor effector response in melanoma immunotherapy. CTLs progressively differentiate from antigen-naïve to terminally differentiated and exhausted, differentiating through effector and memory-like states which contribute to anti-tumor immunity [50]. Terminal differentiation and exhaustion of CTLs is a negative feedback mediated safeguard against uncontrolled immunopathology in the context of resolution of acute immune responses. However, in the context of chronic antigen stimulation and an immunosuppressive TME in cancer, CTL exhaustion is considered maladaptive and leads to failure to control tumor burden. Immune checkpoint blockade works to directly block negative feedback-induced inhibitory signals that curtail CTL activation in the anti-tumor response.

Work from multiple laboratories has demonstrated that a “precursor exhausted” or resource population of CTLs in the tumor-draining lymph node that express both the activation marker PD-1 and stemness marker TCF1 are the key responders to checkpoint blockade in preclinical models of melanoma, colorectal carcinoma and chronic viral infection [51–54]. While these studies suggest immunotherapy can reinvigorate this population, terminally exhausted CTLs within the TME are more refractory to checkpoint blockade in a melanoma model [51]. The transition from precursor exhausted to exhausted is marked by epigenetic changes in LCMV and prostatic adenocarcinoma models, in part mediated by methylation by DNMT3A, notably at the *Tcf7* locus [55]. In a preclinical chronic infection model, treatment with the demethylating agent decitabine prior to ICB leads to enhanced proliferation of CTLs [55]. In a tumor model, de novo methylation at the *Tcf7* and *Ccr7* loci occurs in PD-1^hi^ tumor infiltrating lymphocytes (TILs) relative to PD-1^lo^ TILs, suggesting that exhaustion within the TME is associated with the adoption of methylation programs that in part target these key loci associated with the precursor exhausted state [55].

Features of stemness in precursor exhausted CTLs are epigenetically controlled at multiple levels. In addition to differential direct methylation at the *Tcf7* and other loci, transcriptional control of *Tcf7* and other stemness-associated loci like *Id3*, *Slamf6*, and *Eomes*, trafficking-associated genes like *Ccr7* and *Sell* and anti-apoptotic *Bcl2* is controlled by BACH2, a transcriptional repressor that maintains a chromatin structure associated with stemness in a chronic infection model [56]. Strikingly, BACH2 alters chromatin accessibility predominantly at intronic and intergenic regions, not promoter regions [56]. Using ATAC-seq, the authors showed that the *Bach2* locus itself was epigenetically active in stem-like CTLs [56]. Though some of these findings are from chronic infection models, chronic infection and cancer share some similarities with respect to the nature of chronic antigen stimulation and CTL exhaustion.

CTL exhaustion is also associated with the activity of transcription factor TOX, which has been shown to coordinate with the histone-acetylating HBO1 complex to alter chromatin accessibility in line with an exhausted (Tex) profile in LCMV models [57]. In the context of persistent antigen stimulation in LCMV, Tex cells maintain epigenetic “scarring” that sustains an exhaustion signature even after antigen withdrawal [58]. While advancements in profiling chromatin accessibility have led to insights into the role of global epigenetic changes during CTL differentiation, data on the epigenetic changes specific to direct DNA methylation is more sparse [50].

In addition to the role of DNMT3A in regulating *Tcf7* expression discussed above, the TET family of enzymes play roles in CTL development via conversion of 5mC to 5hmC, resulting in further complete demethylation by TET proteins and base excision repair or demethylation during replication [59, 60]. TET2 is also involved in methylation patterns associated with the CTL memory fate decision in LCMV [61]. Ascorbic acid can synergize with anti-PD-1 therapy in a preclinical lymphoma model in part by increasing cytotoxicity of CTLs associated with an increase in 5mhC levels [62]. In vitro treatment of human CTLs with DAC leads to global demethylation at CpG sites and further epigenetic landscape changes associated with an increase in the expression of short isoforms of NFATc1/A, which is produced in effector CTLs [63]. This DAC-induced change led to increased cytotoxicity in CTLs [63] (Fig. 1). Further studies are needed to elucidate mechanisms by which DNA methylation impacts CTL differentiation in tumors and develop improved drug candidates targeting methylating enzymes that may synergize with immunotherapy in melanoma.

### Epigenetic reprogramming in cell-based therapies

These preclinical studies hold promise for the development of additional epigenetic targeting drugs for combination therapy with immune checkpoint blockade [64]. One challenge in drugging epigenome-modifying enzymes is the potential for off-target effects due to the widespread inhibition of a limited set of enzymes with diverse functions across virtually all cell types with systemic therapy. Precision editing methods (summarized in a subsequent section) as well as engineering cell-based therapies with altered epigenetic profiles hold promise for limiting epigenetic changes only to anti-tumor CTLs.

CAR-T and TCR-T cells are T cells that are engineered to express an antigen receptor (either a chimeric antigen receptor in the case of CAR-T or a TCR in the case of a TCR-T) that is specific for a tumor-associated antigen. The first FDA-approved cell therapy for a solid tumor was the approval of lifileucel for melanoma in 2024. In addition to antigen receptor engineering, cell therapies can be engineered to express additional receptors and signaling modules to further tune their function. Exhaustion is a major limitation for CAR-T and TCR-T function, and modulating the epigenetic landscape of cell-based therapies is an attractive strategy for circumventing exhaustion.

Inadvertent insertion of an anti-CD19 CAR in CAR-T cells for chronic lymphocytic leukemia led to disruption of TET2 [65]. This disruption led to clonal expansion of a memory population of CAR-T, and subsequent evaluation in vitro confirmed that TET2 disruption leads to enhancement of a memory-like population with a distinct effector profile [65]. TET2 disruption led to altered chromatin accessibility at *IFNG, NOTCH2, ICOS*, and other effector-associated genes [65]. A similar mechanism has been shown for the physiologic metabolic byproduct of TCR triggering, S-2-hydroxyglutarate, which regulates DNA methylation and anti-tumor function in CTLs, likely at least in part via the inhibition of TET demethylases [66, 67]. CAR-T treated with the demethylating agent decitabine have enhanced proliferation and anti-tumor function, which was associated with altered methylation at key loci, including *TCF7*, *IL-7R*, *BCL6*, and *EOMES* [68].

The culture and expansion of CTLs for CAR-T and TCR-T therapy can lead to T cell dysregulation mediated by epigenetic changes prior to infusion. In culture, CAR-T cells progressively become hypermethylated at key genes associated with CTL differentiation, including *TOX*, *TCF7,* and *RUNX1* [69]. Finding novel ways of preventing epigenetically associated dysfunction during engineered T cell culture for melanoma immunotherapy is thus critical. Multiple commercial entities are currently investigating the use of epigenetic drugs to “reprogram” T cells to more favorable immunophenotypes with potent tumor killing capabilities.

## DNA methylation as a biomarker for immunotherapy response in melanoma

### Prognostic value of DNA methylation patterns and regulation of immune checkpoints

Technical advancements in accessing the epigenome have generated increased interest in finding biomarkers that could better inform therapy responses in patients. Both epigenetic changes and DNA mutations are hallmarks of tumorigenesis. Epigenetic alterations have been found to play roles in both inducing and sustaining pro-tumorigenic cellular behaviors via several mechanisms [70]. This feature is common across cancer types. Cancers that are highly methylated at genomic sites rich in CpG dinucleotides (CpG islands), which are common in promoters, fall into a distinct phenotypic category characterized by unique histopathological, clinical and prognostic features [71]. This CIMP is found in breast, colorectal, endometrial tumors, leukemias, and glioblastomas [72]. Even in “non-CIMP” cancer methylomes where this characteristic methylation pattern is not seen, general cancer-specific CpG hypermethylation patterns are common [72]. Understanding the etiology and significance of methylation patterns across cancer genomes is thus clinically important for identifying novel prognostic biomarkers, predicting therapy response, and dissecting the mechanisms that direct global methylation changes in cancers.

Specific methylation patterns in melanoma and other cancers have implications for prognosis and treatment. In melanoma, methylation status comprises an independently prognostic parameter. A retrospective analysis of 461 cutaneous melanoma samples from TCGA identified a four-*DNA* methylation signature comprised of four individual methylation sites (cg06778853, cg24670442, cg18456782, cg26263675) associated with overall survival [73]. This signature stratified high-risk from low-risk patients of any Breslow thickness group, correlated with immunotherapy response and performed better than the methylation of TIL (MeTIL) signature, or individual *PD-1*, *PD-L1*, *PD-L2*, and *CTLA-4* signatures [73]. While methylation patterns of tumor cells are highly relevant in prognostication, understanding the methylation patterns of immune cell-specific CpG sites in the tumor microenvironment may also help elucidate mechanisms of immunotherapy responses. As such, an analysis of 180 metastatic melanoma samples with an immune cell-type specific CpG signature found three methylation pattern clusters that correlated with distant metastasis-free survival (DMFS) and melanoma-specific survival (DSS) [74].

The epigenetic signatures at the loci for individual immune inhibitory receptors have also provided prognostic insights into immunotherapy responses. In melanoma, the *PD-L1* promoter is methylated at CpG sites, and *PD-L1* methylation is an independently prognostic biomarker of survival [75]. In anti-CTLA-4 therapy in stage IV melanoma, higher levels of methylation at the *CTLA-4* promoter were associated with progression, while lower levels of methylation were associated with progression-free survival [76]. Acquired resistance to PD-1/PD-L1 monotherapy has also spurred recent developments in alternative immune checkpoint inhibitors. Indeed, immune checkpoint molecules such as TIM3*, LAG3* and *TIGIT* may also be epigenetically regulated and play a role in melanoma prognostication [77, 78]. While an analysis of the TCGA did not identify significant correlations between clinicopathological staging parameters and methylation of CpG sites of *HAVCR2,* its ligand, *LSALS9,* demonstrated methylation of CpG sites correlated with these factors [77]. The authors also demonstrated that DNA methylation status in the promoter regions of *HAVCR2* and *LSALS9* correlated inversely with an IFN-y signature, a cytokine commonly upregulated in successful immunotherapy responses. Similarly, LAG3 is another recently described immune checkpoint molecule. Another analysis of the TCGA cohort [77] had linked *LAG3* promoter hypomethylation to better overall survival in melanoma patients. The authors also tested *LAG3* methylation as a predictive biomarker in 118 immunotherapy-treated melanoma patients and demonstrated a better progression-free survival in patients with hypomethylated melanomas. Low promoter flank methylation of *TIGIT* was prognostic in melanoma patients treated with anti-PD-1 immunotherapy and predicted progression-free survival [79]. Collectively, these studies highlight the significant impact of DNA methylation on immune checkpoint regulation and the potential for improved melanoma prognosis.

## Emerging technologies in melanoma methylation research

### DNA methylation editing

DNA hypermethylation, particularly at the promoter of tumor-suppressor genes, is a well-established epigenetic modification that plays a crucial role in cancer progression. Epigenetic silencing of tumor-suppressor genes impairs normal cellular functions and activates a cascade of events driving cell plasticity and cancer progression [80–82]. Previous attempts at targeted DNA methylation have involved fusing DNMTs to DNA-binding proteins like zinc finger proteins, and transcription activator-like effectors (TALEs) [83–85]. However, designing custom proteins for each specific target sequence is laborious, requiring specialized expertise. Additionally, these studies showed relatively low efficiency of induced DNA methylation at target sites, with significant off-target activity [86]. Pharmacologic DNMT inhibitors broadly regulate DNA methylation in cancer [64] and can cause off-target effects including global hypomethylation, which can potentially activate oncogenes or disrupt normal cellular processes.

The emergence of clustered regularly interspaced short palindromic repeats (CRISPR)-based systems has introduced a powerful toolkit for locus-specific epigenetic manipulation. CRISPR-dead Cas9 (dCas9)-based epigenome engineering has made studying epigenetic perturbations easier, faster, and clinically relevant (Table 2). The dCAS9 endonuclease is directed to specific genomic targets by engineered short guide RNAs (sgRNAs) [64, 87]. Because the sgRNA is the DNA sequence-specific component of the system, it enables efficient targeting of multiple regions, given the ease of designing and synthesizing new sgRNAs. Recent advancements in improving these systems have produced powerful tools that enable precise DNA methylation or demethylation, capable of maintaining epigenetic memory across multiple cell divisions [80, 84, 86–102]. Various CRISPR-based epigenome modifiers are listed in Table 1. A fusion of DNA demethylase Ten-Eleven Translocation (TET) dioxygenase1 (TET1) or DNMT3 with catalytically inactive Cas9 enables targeted DNA methylation editing of methylated or unmethylated promoter sequences leading to activation or silencing, respectively, of the associated endogenous reporter [101]. CRISPR-dCas9-TET1CD has been used to alter expression of the *BRCA1* gene in HeLa and MCF7 cells. DNA demethylation upregulated expression, while methylation led to a reduction in cell growth [80]. Using the SuperNova TAGging (SunTag) system, highly efficacious manipulation of DNA methylation of the *EBF3* gene was shown in multiple melanoma cell lines [91]. *EBF3* is a putative epigenetic driver of melanoma metastasis, which exhibits the paradoxical activation of transcription with a hypermethylated promoter [103]. SunTag involves a repeating peptide array that can recruit multiple copies of an antibody-fusion protein to a specific genomic locus, enhancing targeted DNA methylation [100, 104–106]. This addresses the off-target effects seen with other dCas9-DNMTs, opening the door for studying DNA methylation patterns within melanoma including hypermethylation of tumor-suppressor genes, genes contributing to immune evasion, and discovery of other biomarkers such as ERV expression status [107]. The concept of controlling DNA methylation represents a significant advancement, providing the potential for long-lasting modifications without altering the DNA sequence itself. Unlike direct DNA sequence manipulation, this method reduces the risk of unintended mutations, thereby improving safety. However, off-target DNA methylation can also be detrimental. Therefore, balancing the potential of controlled DNA methylation while avoiding off-target effects is crucial for fully harnessing this approach for therapeutic applications (Table 2).

CRISPRoff is a recently developed programmable epigenetic writer that uses a single dead Cas9 fusion protein to establish DNA methylation and repressive histone modifications in genes and noncoding regions [88]. Transient CRISPRoff expression induces specific DNA methylation and gene repression, which is maintained through cell division and differentiation, potentially enabling targeted epigenetic modulation of the human genome. Importantly, these modifications are reversible with CRISPRon, allowing for the correction of potential complications from CRISPRoff in vivo [88]. As such, CRISPR-based epigenetic engineering could be used to treat a wider range of diseases with epigenetic mechanisms, including melanoma, due to its ability to manipulate specific methylation marks (Jones). Counteracting aberrant epigenetic changes in melanocytes as well as modulating anti-tumor immune responses are areas of further investigation.

#### Developments in genome-wide methylation techniques

The exploration of DNA methylation patterns in melanoma has been significantly advanced by the development of genome-wide methylation assays. Bead chips involve hybridizing bisulfite-converted DNA to probes that target specific CpG sites across the genome. The earliest platforms allowed detection of methylation changes at ~ 27 K CpG sites, mostly promoter regions and known cancer genes [108]. In 2011, an updated beadchip interrogated methylation status of over 450 K CpG sites and became a widely popular platform for epigenome-wide association studies [108]. The latest iterations allowed targeting of almost double the probe content (~ 860 K probes), including cis-regulatory elements, and lately the HumanMethylationEPICv2 in 2023 with coverage of over 900 K sites, including CpG islands, enhancer regions and open chromatin sites [109]. The genome-wide beadchip arrays were used in the TCGA project, which utilized them to characterize the methylation profiles of cutaneous melanoma samples and correlate these with clinical outcome parameters [110]. This workhorse assay has been used to generate useful datasets for studying methylation melanoma, including the comparison of responders and non-responders to checkpoint therapies [111].

However, even at over 900 K probes covered, this represents only a fraction of ~ 28 million CpG sites in the human genome. There may also be underrepresentation of certain genomic regions like repetitive elements and limitations in the efficiency of bisulfite conversion if working with low amount of DNA from rare immune cells and GC bias. An alternative approach that overcomes bisulfite conversion is consecutive enzymatic treatment with TET2 and APOBEC followed by DNA sequencing. Using this enzymatic approach, unmethylated cytosines are converted to uracil and 5mc is oxidized to 5caC, which end up being sequenced as thymine and cytosine, respectively. Using this approach, it is possible to achieve improved coverage and more even representation while reducing GC bias.

Single-cell sequencing has transformed our understanding of tumor heterogeneity and the tumor microenvironment (TME). By examining the methylation profiles of individual cells within the TME, researchers can identify diverse cell populations and reveal mechanisms of drug resistance. Single-cell bisulfite conversion sequencing (BS-seq) has enabled high-quality DNA methylation profiling across the entire epigenome [112, 113]. This has recently been made possible by enhancing the recovery of bisulfite-converted DNA and addressing issues with degradation and fragmentation [114, 115]. Various methods using BS-seq have been developed including single-cell whole-genome bisulfite sequencing (scWGBS), reduced-representation bisulfite sequencing (RRBS), and single-cell combinatorial indexing methylation sequencing (sci-MET) [112, 116, 117]. scWGBS allows for high-resolution whole-genome methylation profiling at the single-cell level, but is labor-intensive and less scalable. DNA is treated with sodium bisulfite, which converts unmethylated cytosines into uracil, while methylated cytosines remain unchanged. RRBS enriches for CpG-rich regions, making it a more cost-effective but less comprehensive technique [117, 118]. sci-MET uses combinatorial indexing to profile DNA methylation in thousands of individual cells in a single experiment, allowing for high-throughput single-cell methylation analysis [116]. Methylation can also be assessed using conversion-free methods, but only genome-wide CpG island methylation sequencing for single cells (scCGI-seq) is scalable to the single-cell level [89]. It involves a methylation-sensitive restriction enzyme digestion, followed by multiplexed displacement amplification, which allows for the genome-wide measurement of methylation in CpG-rich regions in single cells [119]. The development of these techniques has allowed for the further integration of methylation, transcriptome and chromatin accessibility profiling in single cells (scNMT) [113, 120]. These high-resolution methods could support the development of more precise, personalized treatments for melanoma [121]. However, single-cell methylation approaches face the same challenges of cost, technical complexity, and the need for specialized bioinformatics, along with requiring fresh or well-preserved tissue samples [89, 112].

## Conclusions

The success of immunotherapy has revolutionized the treatment of melanoma. However, only about half of patients have durable responses. Recent advances in understanding how epigenetic changes in tumor cells and immune cells in the TME shape the anti-tumor response have spurred interest in finding epigenetic targets that could potentiate and broaden the scope of immunotherapy in melanoma.

Epigenetic changes are key drivers of both tumorigenesis and immune cell development in the TME. DNA methylation in particular has been shown to play key roles in the modulation of tumor suppressor, oncogene, and ERV expression in tumor cells. DNA methylation further shapes the phenotypic trajectory of CTLs in the anti-tumor response, modifying the expression of key stemness and exhaustion genes. Targeting these mechanisms has been promising, as treatment with DNMTis has synergized with immune checkpoint blockade in preclinical studies. However, targeting DNMTs is pharmacologically challenging [122] and requires further research to generate novel drug candidates.

Advances in methylation techniques have enabled unprecedented insights into changes in methylation across the genome at the single-cell level. Continued advancements in integration of single-cell methylation techniques with other single-cell profiling techniques will provide mechanistic insights into the coordinated and sequential control of tuning of gene expression in tumor and immune cells across the anti-tumor response. These developments will further enable a higher resolution understanding and identification of potential biomarkers that correlate with immunotherapy response in melanoma. Further advancements in understanding how methylation contributes to immunotherapy responses in melanoma, development of methylation editing tools and methyltransferase drug development have significant implications for improving the care of patients with melanoma.

**Table 1:** Summary of CRISPR-based DNA methylation editing systems for mammalian cells

System	Effect	Mechanism
dCas9-DNMT3A	gene repression	DNMT3A facilitates de novo DNA methylation, repressing the target gene [86, 91, 99]
dCas9-DNMT3A3L	gene repression	DNMT3L boosts DNMT3A methylation activity despite itself lacking a catalytic domain, increasing system efficacy [102]
dCas9-DNMT3A3L-KRAB	gene repression	The KRAB domain is added to the dCas9 system to enhance repression beyond DNMT3A and DNMT3L alone [123]
dCas9-MQ1	gene repression	MQ1, an engineered prokaryotic CpG DNA methyltransferase, is fused to dCas9, enhancing methylation activity [124]
dCas9-sMTase	gene repression	A “split methyltransferase” derived from *M.SssI* is used, where the two components bind at the target CpG site [96]
dCas9-scFv-DNMT3A (SunTag-DNMT3A)	gene repression	The SunTag system is used with scFv-DNMT3A (instead of VP64) to recruit multiple DNMT3A proteins to the target region [106]
dCas9-scFv-DNMT3AL (SunTag-DNMT3AL)	gene repression	The SunTag system is used with scFv-DNMT3AL (instead of VP64) to recruit multiple DNMT3AL proteins to the target region [125]
dCas9-SunTag system	gene activation	The SunTag protein is linked to dCas9, with VP64-fused scFv added to recruit multiple VP64 copies, enhancing gene activation beyond dCas9-VP64 alone [126, 127]
dCas9-TET1	gene activation	TET1 removes methyl groups to activate transcription. The dCas9-TET1 system enables targeted DNA demethylation and gene activation [80, 127]
dCas9, Tet1-MS2	gene activation	This system uses Effector-MS2 to recruit multiple TET1 proteins, increasing system efficacy [98]
dCas9-scFv-TET1 (SunTag-TET1)	gene activation	This system uses SunTag with scFv-TET1 fusion (instead of VP64) to recruit more TET1 proteins, enhancing DNA demethylation [97]
*Casilio*-ME	gene activation	The *Casilio* platform is used to co-deliver TET1 and BER-associated proteins GADD45A or NEIL2 for a higher efficacy than SunTag systems [128]
dCas9-R2	gene activation	The dCas9-R2 module specifically binds and inhibits the action of endogenous DNMT1 to prevent local DNA methylation at the target site [129]
Dual Cas9, Template	gene repression or activation	Excised a 1120 bp CGI from the *HPRT1* promoter using dual gRNA-guided Cas9 and replaced it with fully methylated or unmethylated fragments via NHEJ [130]
dCas9-ROS1	gene activation	ROS1, a plant-specific DNA glycosylase, directly excises 5mC [131]
CRISPRoff	gene repression	A novel dCas9-DNMT3A-3LZNF10 KRAB system leads to heritable, persistent gene silencing [88]
CRISPRon	gene activation	dCas9-TET1 and modified sgRNA with MS2 stem-loop sequences interact with MS2 coat protein fused to the VPR system, to activate silenced genes [88]

**Table 2:** Summary of DNA methylation editing and detection techniques

Technique	Principle	Advantages	Limitations	Applications in Research
CRISPR-dCas9 methylation editing	Catalytically inactive Cas9 (dCas9) fused to DNMT or TET enzymes for targeted methylation or demethylation; SunTag enhances targeting efficiency via peptide arrays	Precise locus-specific targeting; easy sgRNA design; enhanced efficiency using SunTag; maintains epigenetic memory	Potential off-target methylation; complexity in SunTag optimization	Precise epigenetic editing; functional studies of gene regulation; modeling tumor suppressor and oncogene expression
CRISPRoff/CRISPRon	Programmable, reversible epigenetic modulation (DNA methylation and histone modifications) using dCas9 fusion proteins	Stable and reversible epigenetic changes; minimal risk of DNA mutations	Potential off-target effects; requires further in vivo validation	Studying reversible epigenetic regulation; exploring therapeutic potential for diseases involving aberrant gene silencing or activation
Genome-wide beadchip arrays (HumanMethylationEPICv2)	Bisulfite-converted DNA hybridized to genome-wide CpG probes (> 900 K sites)	Extensive coverage; validated, cost-effective method for genome-wide methylation profiling. Widely available open access bioinformatic packages for analysis	Limited genome-wide coverage (~ 28 M total CpGs); poor representation of repetitive regions; potential GC bias	Epigenome-wide association studies; biomarker discovery; correlating methylation profiles with clinical outcomes and therapeutic responses
Enzymatic methylation sequencing (TET2/APOBEC-based)	TET2 and APOBEC enzymes discriminate methylated from unmethylated cytosines without bisulfite-induced degradation	Improved genome-wide coverage; reduced GC bias; more uniform representation of methylation sites	Requires careful enzymatic optimization; bioinformatic complexity	Comprehensive profiling of DNA methylation; improved assessment of methylation patterns in diverse cell types, including rare immune cells
Single-cell bisulfite sequencing (scWGBS, RRBS, sci-MET)	Single-cell resolution DNA methylation analysis via bisulfite conversion	High-resolution methylation profiling; reveals cellular heterogeneity; detects rare or transient cell states	Technically demanding; expensive; requires fresh tissue and specialized bioinformatics tools	Characterizing tumor and tissue heterogeneity; exploring mechanisms of drug resistance; integration with other single-cell “omics” approaches
Single-cell CGI-seq (scCGI-seq)	Conversion-free method using methylation-sensitive restriction enzymes for single-cell methylation profiling	Scalable; avoids DNA damage associated with bisulfite treatment; suited for CpG island methylation	Limited to CpG-rich regions; intermediate genome coverage	Profiling methylation in CpG islands at single-cell resolution; integration into multi-omics single-cell workflows

## Data Availability

No datasets were generated or analyzed during the current study.

## Abbreviations

TIL: Tumor-Infiltrating Lymphocytes
ACT: Adoptive Cell Transfer
ICI: Immune Checkpoint Inhibitor
ORR: Objective Response Rate
PD-1: Programmed Cell Death Protein 1
CTLA-4: Cytotoxic T-Lymphocyte Associated Protein 4
NSG: NOD scid gamma (mouse model)
PDX: Patient-Derived Xenograft
IL-7R: Interleukin-7 Receptor
MHC: Major Histocompatibility Complex
ERV: Endogenous Retrovirus
5-AZA-CdR: 5-Aza-2′-deoxycytidine (decitabine)
TET1: Ten-Eleven Translocation 1
CRISPR: Clustered Regularly Interspaced Short Palindromic Repeats
scRNAseq: Single-Cell RNA Sequencing
dCas9: Dead Cas9
DNMT3A: DNA Methyltransferase 3 Alpha
DNMT: DNA Methyltransferase
CpG: Cytosine-phosphate-Guanine
ATAC-Seq: Assay for Transposase-Accessible Chromatin using Sequencing
5mc: 5-Methylcytosine
IL-2: Interleukin-2
Bcl6: B-Cell Lymphoma 6
HD IL-2: High-Dose Interleukin-2
PD-L1: Programmed Death-Ligand 1
DAC: Decitabine
